# The role of high-sensitivity troponin in identifying patients with cardiac allograft rejection

**DOI:** 10.1016/j.jhlto.2025.100446

**Published:** 2025-12-03

**Authors:** André A. Scussel, Fabiana G. Marcondes-Braga, Cristhian V. Espinoza Romero, Daniel C. de Marchi, Mônica Samuel Ávila Grinberg, Fernanda Barone Alves do Santos, Ana Maria Peixoto Cardoso Duque, Sandrigo Mangini, Luís Fernando Bernal Seguro, Iáscara Wozniak Campos, Fabio Gaiotto, Fernando Bacal

**Affiliations:** Heart Institute (InCor), University of São Paulo Medical School, São Paulo, Brazil

**Keywords:** Heart transplantation, Acute cellular rejection, Cardiac biomarkers, High-sensitivity troponin, Endomyocardial biopsy

## Abstract

**Background:**

Biomarkers for acute rejection diagnosis are frequently searched, and data from medical literature are conflicting. The role of high-sensitivity troponin I in the diagnosis of acute cellular rejection (ACR) after heart transplant (HT) is uncertain. However, it is a widely used and accessible tool in developing countries.

**Objective:**

This study aims to determine whether hs-TnI can serve as a reliable and accessible tool to identify patients with clinically significant rejection (ACR >2R) in a real-world Latin American cohort that has different etiologies than the epidemiology of the main trials.

**Methods:**

In this retrospective cohort, we evaluated data from electronic records of HT recipients submitted to HT from March, 2020 to September, 2022 using REdCAp database. All patients who underwent endomyocardial biopsies (EMB) between 3 months and 2 years of HT and had samples of HS-troponin I previously measured were included in this study. The HS-troponin levels were compared between patients with ACR higher or lower than 2R.

**Results:**

In this analysis, we included data from 187 biopsies performed at least 3 months after HT in 94 recipients who had paired Hs-troponin I samples collected. Fifty-four (57%) were men, and their median age at heart transplant was 48 ±11 years. The median hs-TnI levels were 29 (11-118) ng/L. ACR > 2R was observed in 48 EMB and the median hs-TnI levels were 69 (26-224) ng/L, which was significantly higher than levels observed in patients with grade 0R/1R [21 (9-64) ng/L, *p*<0.001]. The ROC curve of hs-TnI shows an AUC of 0.705; *p*<0.001 for the diagnosis of ACR > 2R. A cutoff of 19 ng/L showed a sensitivity of 88% and specificity of 49%, with a negative predictive value of 92%. Among biopsies from patients with Chagas disease (36.9% of the sample), hs-TnI also showed significant discriminatory power for ACR >2R (*p* = 0.037).

**Conclusion:**

In this cohort, HS-troponin I was able to identify patients with acute cellular rejection. This is the largest Latin American cohort assessing hs-TnI after HT, and the first to explore its performance across specific etiologies, including Chagas disease. Prospective studies may confirm the role of HS-troponin I as a biomarker of acute rejection.

Heart transplantation (HT) has become a life-saving therapeutic option for patients with end-stage heart failure (HF). However, acute cellular rejection (ACR) remains a significant concern in the first year following transplantation, affecting approximately 30% of recipients and often presenting with asymptomatic features.^1^ Early recognition and timely treatment of ACR are crucial to improve long-term outcomes and graft survival.^1^ Currently, endomyocardial biopsy (EMB) serves as the gold standard for diagnosing ACR.^1^, ^2^ Despite its diagnostic accuracy, EMB is an invasive procedure with associated risks and limitations as an interobserver variability by pathologists and tricuspid valve lesions.^3^, ^4^ The need for less invasive diagnostic tests with reasonable predictive capacity has led to the investigation of alternative biomarkers.

Among the noninvasive tests assessed, the gene expression profile (Allomap) has demonstrated promising results for ACR surveillance, comparable to EMB. However, the practicality of Allomap is restricted by its low positive predictive value, high cost, and time-consuming process.^5^, ^6^ Another method for ACR monitoring is the measurement of donor-derived cell-free DNA, which can assist in detecting both ACR and antibody-mediated rejection (AMR).^16^ However, it has similar practical limitations in terms of cost and availability.

The role of high-sensitivity troponin (hs-TnI) as a potential diagnostic biomarker for various cardiovascular conditions has gained considerable attention.^7^ Cardiac troponins I and T, sarcomeric structural proteins, are released into the bloodstream following myocardial injury and cardiomyocyte damage.^7^ With the advent of hs-Tn assays, boasting 10 times greater sensitivity than conventional tests, hs-Tn exhibits favorable sensitivity and negative predictive value.^2^ For this reason, the recent guidelines of the International Society of Heart and Lung Transplantation (ISHLT) recommend the integration of biomarkers such as high-sensitivity troponin into a rejection monitoring strategy to identify higher-risk patients who may benefit from further evaluation for ACR.^8^, ^9^ This study aims to determine whether hs-TnI can serve as a reliable, accessible tool for identifying patients with clinically significant rejection (ACR >2R) in a real-world Latin American cohort. The high sensitivity and negative predictive value of hs-TnI offer promising prospects for a noninvasive and reliable diagnostic tool, enabling early identification and management of ACR in patients with HT.

## Methods

### Study design, setting, and participants

This is a single-center retrospective cohort study in which we evaluated data from electronic records of patients of our institution that were submitted to HT between March 2020 and September 2022. We analyzed values of hs-TnI measured until 48 hours before EMB and their association with ACR incidence. Evidence suggests that biomarker measurements within the first 3 months postheart transplant lack reliability due to a high incidence of false positives. This can be attributed to the continuous release of troponin observed over a 2 to 3 months period following heart transplantation.^16^ Therefore, biopsies performed before 3 months of heart transplant were excluded. The flowchart of the study is represented below.

### Statistical analysis

All statistical analyses were performed using SPSS for Windows, version 26.0. Categorical variables were expressed as absolute (n) and relative frequencies (%), and were compared using the Pearson’s Chi-square test. Numerical variables were initially tested for normality using the Kolmogorov-Smirnov test with Lilliefors correction to assess normality.

Variables with normal distribution were presented as mean ± standard deviation (SD) and compared between groups using the independent samples Student’s t-test. Non-normally distributed variables were expressed as median and interquartile range (IQR), and compared using the Mann-Whitney U test.

To evaluate the diagnostic accuracy of troponin levels in predicting rejection, receiver operating characteristic (ROC) curve analysis was performed. The area under the ROC curve (AUC) was calculated to assess sensitivity, specificity, and overall diagnostic performance.

The institutional ethical committee approved the study (CAAE:42622215.9.0000.0068).

## Results

In this analysis, we included data from 187 biopsies performed at least 3 months after HT in 94 recipients who had paired Hs-troponin I samples collected. Clinical data are shown in Table 1.

**Table 1:** Clinical and Demographic Data of Heart Transplant Patients in This Cohort

Patients’ characteristics (n = 94)	Value
Age at heart transplant (y)	48.0 ± 10.7
Male sex n (%)	54 (57.4)
Etiology of Cardiomyopathy (n [%])	
Dilated	41 (43.6)
Chagas Disease	34 (36.2)
Ischemic	8 (8.5)
Other	11 (11.7)
Donor age (y)	29.8 ± 9.5
Donor male sex (%)	72 (76.6)
Heart transplant in priority condition (n [%])	
Total	93 (98.9)
Inotropes	47 (50.0)
IABP	40 (42.6)
Other	6 (6.4)

CM, cardiomyopathy; IABP, intraaortic balloon pump. Numerical variables are expressed as mean ± standard deviation or median and interquartile range, depending on the result of the normality test.

**Figure 1:**
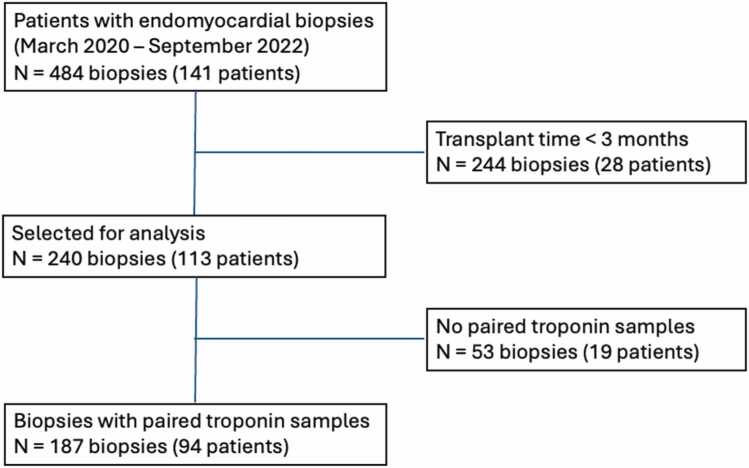
Flowchart of the study

The median hs-TnI levels were 29(1(11−118)g/L. ACR >2R was observed in 48 EMB and the median hs-TnI levels were 69(2(26−224)g/L, which was significantly higher than levels observed in patients with grade 0R/1R [21(9 (9−64)g/L, *p*<0.001 (Figure 1).

The characteristics of patients with treated rejection (EMB >2R) or no rejection did not differ in terms of ejection fraction or the presence of RV dysfunction. However, we observed a higher level of serum creatinine in the group of no rejection (Table 2).

**Table 2:** Characteristics of Patients According to Acute Cellular Rejection Group

Variables	EMB <2R (n = 48)	EMB ≥2R (n = 139)	*p*
Age (y)	49 (40-56)	50 (36-56)	0.889
Echocardiogram findings			
LVEF (%)	61 (55-65)	62 (60-65)	0.197
RV dysfunction (%)	15 (10.8)	7 (14.6)	0.482
Lab tests			
HS-troponin I (ng/L)	21 (11-118)	69 (26-224)	<0.001
Serum Creatinine (mg/dl)	1.50 (1.14-2.03)	1.30 (1.08-1.68)	0.032

Numerical variables are expressed as mean ± standard deviation or median and interquartile range, depending on the result of the normality test.

**Figure 2:**
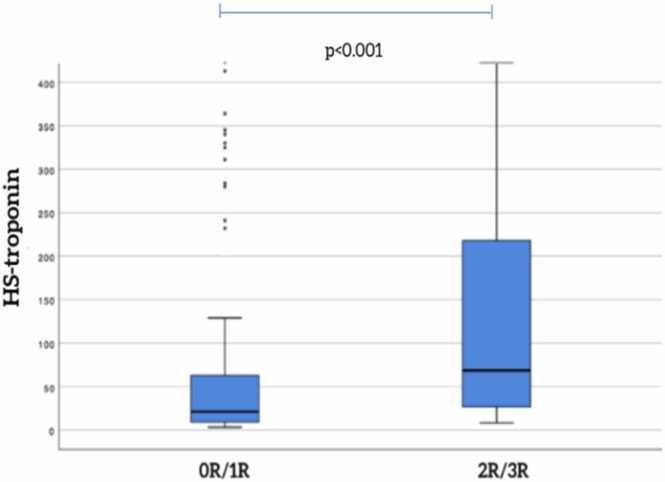
Hs-TnI levels according to rejection grade

**Figure 3:**
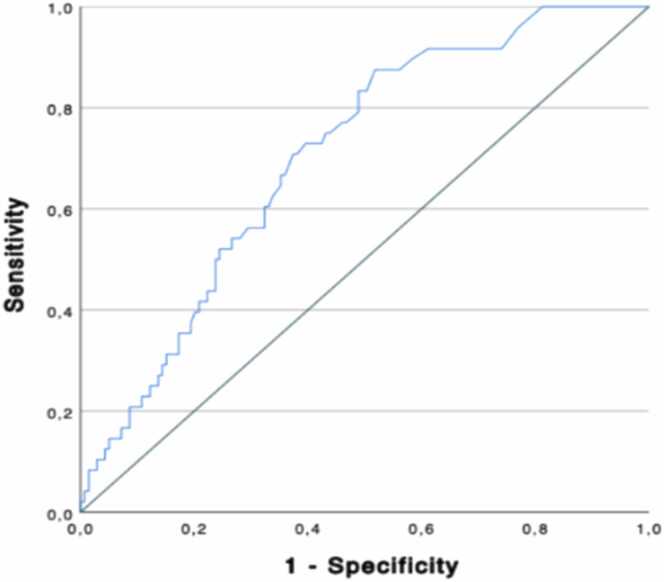
ROC curve for hs-TnI in predicting rejection

**Figure 4:**
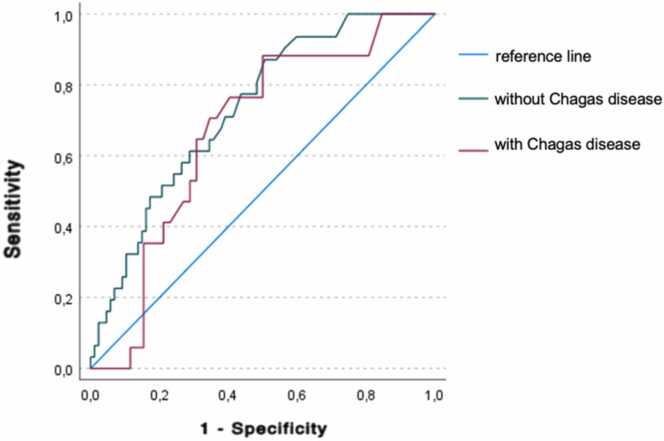
ROC curve for hs-TnI in predicting rejection in Chagas disease subgroup

The ROC curve (depicted in Figure 2) for HS-troponin I indicates an AUC of 0.705, with a *p*-value of <0.001 for diagnosing ACR ≥2R. Using a cutoff value of 19 ng/L, hs-TnI yielded a sensitivity of 88% and a specificity of 49%, corresponding to a negative predictive value of 92% in this population. Figure 3, Figure 4.

The performance of hs-TnI was also analyzed in specific subgroups, including patients with Chagas disease, a population often underrepresented in heart transplant studies. Among these patients, hs-TnI levels were significantly higher in biopsies with ACR >2R [42 ng/L (28-(28−72) compared to those with grade 0R/1R [23 ng/L (11-(11−46) = 0.037). The ROC curve in this subgroup showed an AUC of 0.669 (*p* = 0.016). No statistically significant difference was observed between the ROC curves of patients with and without Chagas disease (Z-test, *p* = 0.464).

## Discussion

The main findings of the current study were

(i) The hs-TnI levels in patients with ACR >2R were significantly higher than levels observed in patients with grade 0R/1R;
(ii) A cutoff value of 19 ng/L for hs-TnI demonstrated a sensitivity of 88% and a specificity of 49% for diagnosing ACR;
(iii) hs-TnI levels were also significantly elevated in patients with ACR >2R within specific subgroups, such as those with Chagas disease.

The high incidence of ACR during the first posttransplant year underscores the need for reliable diagnostic methods with minimal invasiveness.^10^ However, biomarker measurements within the first 3 months after the procedure may lead to false positives.^10^ To address this issue, we reviewed 2 recent meta-analyses, involving a total of 993 and 840 patients, respectively, with significant cellular rejection (defined as ≥2R).^2^, ^11^ These studies evaluated the use of conventional troponin (TnC) and hs-TnI in diagnosing ACR. While the use of hs-TnI showed a higher sensitivity and negative predictive value compared to TnC, it also exhibited some limitations, such as high heterogeneity and retrospective nature of some studies.^2^, ^11^ Further investigations showed that hs-TnI concentrations were significantly higher in rejection versus nonrejection samples, demonstrating their potential as a diagnostic marker.^11^ In a retrospective study of 98 patients, hs-TnI showed a sensitivity of 94% and negative predictive value of 99% when adjusted for a cutoff of 15 ng/L.^12^ Similar results were obtained in another study with a sensitivity of 80% and a negative predictive value of 96%.^13^

In comparison to noninvasive methods like gene expression profiling (AlloMap), hs-TnI demonstrated excellent negative predictive value and sensitivity in appropriate low-risk patients.^14^ Although those tools have shown good performance in rejection surveillance, their high costs limit their use, particularly in low-resource settings. In such scenarios, protocols incorporating hs-TnI in selected low-risk patients — ideally within a multimodal approach that includes BNP measurement and transthoracic echocardiography — may help reduce the need for EMB or gene expression profiling.^14^, ^15^

While similar studies have been conducted in other populations, this is the first and largest to investigate heart transplant recipients in a Latin American cohort, which presents unique characteristics such as a higher prevalence of Chagas disease. In Latin America in general, access to advanced diagnostic tools—such as gene expression profiling or donor-derived cell-free DNA—is often limited due to economic and structural constraints. In this context, hs-TnI may represent a promising and accessible biomarker to support the identification of patients at higher risk of rejection for whom prioritized EMB may be warranted, used in conjunction with other clinical and echocardiographic parameters.

We also explored whether the few biopsies with grade 3R rejection exhibited disproportionately higher hs-TnI levels; however, the number of 3R events was extremely small, preventing meaningful statistical analysis.

## Limitations

This study presents some limitations. First, it is a single-center retrospective cohort, which may limit the generalizability of the results. The analysis was based on data extracted from electronic medical records, which are subject to potential recording bias and missing information. Additionally, although the total number of biopsies was considerable, the number of events with rejection >2R was relatively small, which may reduce the statistical power of the comparisons.

## Conclusions

Our study described the potential of hs-TnI as a valuable biomarker for diagnosing ACR posttransplantation. Incorporating hs-TnI into posttransplant surveillance strategies may improve early rejection detection and patient outcomes, particularly in Latin American settings and other regions where access to advanced noninvasive diagnostic tools is limited. Moreover, this study highlights the utility of hs-TnI in patients with Chagas cardiomyopathy—a population often underrepresented in transplant literature. Further research and clinical trials are warranted to validate and optimize the use of hs-TnI in routine clinical practice, ultimately improving patient care and outcomes.

## Disclosure statement

The authors declare no conflicts of interest.

## Declaration of Generative AI and AI-assisted technologies in the writing process

During the preparation of this work, the author(s) used ChatGPT (OpenAI) to improve language and readability, with caution. After using this tool/service, the author(s) reviewed and edited the content as needed and take(s) full responsibility for the content of the publication.

## Declaration of competing interest

The authors declare that they have no known competing financial interests or personal relationships that could have appeared to influence the work reported in this paper. We have nothing to disclose.
